# Discreditable

**Published:** 1869-08-15

**Authors:** 


### *Discreditable.*

Among the ablest of our exchanges is the *Richmond & Louisville Medical and Surgical Journal*, edited by Prof. E. S. Gaillard, M.D., one of the most industrious students, accomplished and versatile of writers, connected with the profession. It seems that in the course of his usual work upon the *Journal*, he had occasion to review a production by one Bell, who we believe is connected with some college or school south of the Ohio river. This review contained nothing but what was in every way legitimate and proper in journalistic criticism, whatever discrepancy of view might be indulged as to the subject matter. But this Bell took the thing in the highest dudgeon. If ever mellifluous in expression, we can assure him that he has broken into a harshness and dissonance which augurs that he has been *cracked* to utter noise *et preterea nihil.* Although we do not know him from Adam (after the fall), and never heard of him save in the present half-Thersites, half-Billingsate tones, nevertheless, from the depths of the kindness which notably warms the editorial heart, we urge upon him, for his own well-being, that he betake himself incontinently to silence, or to some seven-times heated furnace of discipline, where he may repair damages, and no longer air his discreditable talk to the immediate danger of all tympani. Let us have peace!

